# Diagnostic models of the pre-test probability of stable coronary artery disease: A systematic review

**DOI:** 10.6061/clinics/2017(03)10

**Published:** 2017-03

**Authors:** Ting He, Xing Liu, Nana Xu, Ying Li, Qiaoyu Wu, Meilin Liu, Hong Yuan

**Affiliations:** I Central South University, The Third Xiangya Hospital, Department of Cardiology, Changsha 410013; II Central South University, The Third Xiangya Hospital, Center of Clinical Pharmacology, Changsha 410013; III The First Hospital of Beijing University, Department of Gerontology, Beijing, The People’s Republic of China

**Keywords:** Coronary Artery Disease, Risk Assessment, Pre-Test Probability, Systematic Review, Diagnosis

## Abstract

A comprehensive search of PubMed and Embase was performed in January 2015 to examine the available literature on validated diagnostic models of the pre-test probability of stable coronary artery disease and to describe the characteristics of the models. Studies that were designed to develop and validate diagnostic models of pre-test probability for stable coronary artery disease were included. Data regarding baseline patient characteristics, procedural characteristics, modeling methods, metrics of model performance, risk of bias, and clinical usefulness were extracted. Ten studies involving the development of 12 models and two studies focusing on external validation were identified. Seven models were validated internally, and seven models were validated externally. Discrimination varied between studies that were validated internally (C statistic 0.66-0.81) and externally (0.49-0.87). Only one study presented reclassification indices. The majority of better performing models included sex, age, symptoms, diabetes, smoking, and hyperlipidemia as variables. Only two diagnostic models evaluated the effects on clinical decision making processes or patient outcomes. Most diagnostic models of the pre-test probability of stable coronary artery disease have had modest success, and very few present data regarding the effects of these models on clinical decision making processes or patient outcomes.

## INTRODUCTION

Coronary artery disease (CAD) is a common public health problem that is frequently associated with high mortality and increased health costs. Invasive coronary arteriography (ICA), the gold standard procedure for diagnosing CAD, has been widely used in clinical practice. Although this procedure can reduce the misdiagnosis of CAD in patients, it may also be an excessive medical treatment. In a recent report, only 41% of patients who underwent ICA were then diagnosed with obstructive CAD, suggesting that at least half of the people who undergo this expensive procedure do not need it 1. Models that can predict the pre-test probability (PTP) of stable CAD in patients may serve as an effective “gatekeeper” to identify those who are at a high risk and who may benefit from further diagnostic investigation.

The European and American guidelines have recently placed great importance on the initial risk stratification of suspected CAD to avoid unwarranted examinations and have recommended the Duke clinical score (DCS) and Diamond-Forrest model (DFM) as the preferred models to calculate the PTP 2,3. However, some studies have suggested that these two risk assessment methods perform poorly in Asian populations, especially in China 4,5. Indeed, differences in derivation, inconsistent external validation, and complexity of the models often exist, which limit their general application in daily clinical practice. In addition, the effects of adopting clinical prediction rules to guide decision making and improve patient outcomes are often not evaluated. Therefore, we performed a systematic review of the available literature on validated diagnostic models of PTP for obstructive CAD and described the performance of the models and their clinical utility to better understand the development of diagnostic models and to help clinicians select the ideal model to use in their practices.

## METHODS

### Literature search

We conducted a systematic review of the available literature using the Preferred Reporting Items for Systematic Reviews and Meta-Analyses (PRISMA) guidelines 6. PubMed and Embase were searched in January 2016 by professional document retrieval personnel from the Xiang-Ya Medical Library of Central South University using the following search terms: ‘coronary heart disease,’ ‘coronary artery disease,’ ‘stable angina,’ ‘pre-test probability,’ and ‘probabilistic model.’ Reference lists in relevant meta-analyses and reviews were manually examined. No language or data restrictions were included. All of the potentially relevant studies were examined in their entirety.

### Selection criteria

Clinical prediction models can often be divided into diagnostic models that estimate the probability of a specific diagnosis and prognostic models that estimate the probability of expected outcomes over a given period of time 7. In patients with suspected CAD, diagnostic models can provide a method to calculate the PTP, whereas prognostic models, such as the Framingham risk score, PROCAM (Prospective Cardiovascular Munster) score, and SCORE(Systematic Coronary Risk Evaluation) score, are used to stratify risk and to predict the clinical outcomes but not the anatomical results. In addition, prognostic models are often developed using asymptomatic patients 8,9. In our review, we mainly focused on diagnostic models.

We included articles if they evaluated the characteristics of a diagnostic model of CAD, regardless of whether they included information about the development or validation of the models. The inclusion criteria were as follows: 1 the model contained at least two independent variables; 2 the eligible clinical endpoint was a stable CAD; and 3 because predictive factors that are derived in a single population could lack validity and applicability, we included only studies that presented both the development and validation of the diagnostic model. We did not specify the method of validation in advance, nor did we exclude studies where the derivation and validation cohorts (possibly from other studies) were drawn from the same population. The exclusion criteria were: 1 the model was developed using patients with acute coronary syndrome, unstable chest pain, a history of myocardial infarction, or previous revascularization (percutaneous coronary intervention or coronary artery bypass graft surgery); 2 the clinical endpoint was myocardial infarction or death; or 3 the article was an unpublished conference abstract.

### Data extraction

Two reviewers (Ting H and Xing L) extracted data from the included studies. The following items were recorded in standardized forms: the names of the authors, publication year, definition used for CAD, sample size, and method used for model development. We also noted the variables included in the model and the geographic origin of each study. If an article described more than two models, we considered all of the possible models as eligible for inclusion in our review. We also extracted metrics of model performance (discrimination, calibration, and reclassification).

Correspondence with the authors of the included studies was initiated as necessary. If discrepancies occurred, a consensus was reached by all authors through discussion. All of the obtained data were carefully examined for accuracy.

### Model performance and validation

We evaluated the internal validity of each model by examining its discrimination power, calibration, and reclassification. The traditional metric used to assess model discrimination, the C statistic (equivalent to the area under the receiver operating characteristic curve), was extracted. This value represented the model’s ability to distinguish patients with and without CAD. Typically, modest discriminative ability was defined by C statistic values that ranged from 0.7 to 0.8, whereas values that were greater than 0.8 were indicative of good discriminative ability. Other metrics, such as sensitivity and specificity, were included if the C statistic was not reported.

To determine the calibration, we used the Hosmer-Lemeshow statistic, which was used to verify that the degree of the average prediction was consistent with the observed outcome. A larger p-value indicated better calibration ability (a *p*-value>0.1 was considered evidence of adequate calibration). If the Hosmer-Lemeshow statistic was not reported, we extracted information on the calibration plot, if shown.

Reclassification was evaluated using the net reclassification improvement (NRI). The NRI compares the frequency with which appropriate reclassification occurs to the frequency with which inappropriate reclassification occurs in the use of the new model 10. For this test, a value of *p<0.05 suggested that a significantly greater number of patients were being reclassified appropriately than were being reclassified inappropriately.

We also evaluated the generalizability of each diagnostic model by determining whether it had been externally validated in an independent patient population, either in the original study or in a subsequent publication.

### Quality assessment and clinical usefulness

The methodological quality and risk of bias of each model were evaluated by two reviewers in accordance with the criteria recommended by Hayden and colleagues 11,12. The criteria were as follows: study participation, study attrition, prognostic factor selection, prognostic factor measurement, outcome measurement, statistical analysis, and reporting of model performance (discrimination, calibration, and reclassification). All of the items were assigned a value of ‘low risk,’ ‘high risk,’ or ‘uncertain.’

We also assessed the clinical usefulness of each model, which was defined as the combination of clinical utility and usability. For clinical utility (the effect on a clinical decision that is linked to a risk category or threshold), we determined whether the authors linked their models to specific risk categories and discussed how the risk categories would aid diagnostic evaluations. For usability (the availability of a clinical decision tool), we noted whether the authors included a calculator or risk score that would facilitate knowledge translation and use at the bedside.

## RESULTS

### Included studies

A total of 1333 studies were identified through PubMed and Embase searches. After removing duplicate studies, 1175 abstracts were screened. A total of 26 relevant full-text articles were assessed for eligibility, of which 12 trials comprising 12 unique risk diagnostic models fulfilled the inclusion criteria.

**Figure 1:**
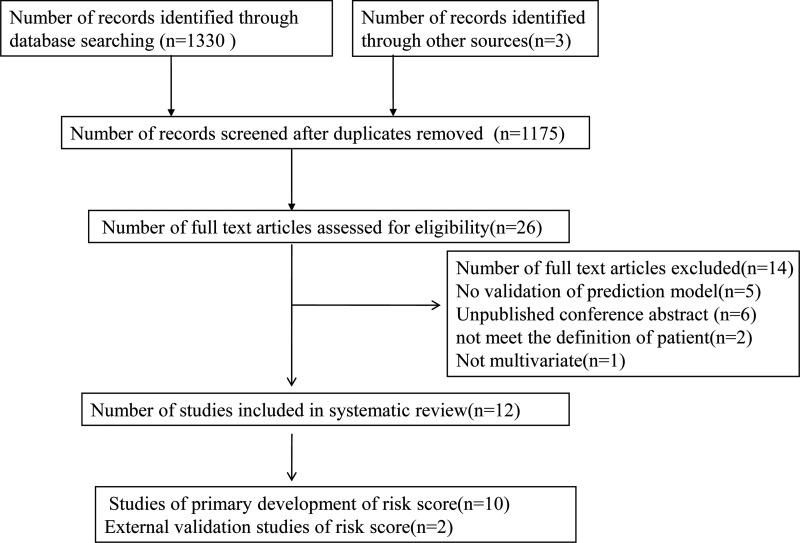
Flow chart for eligible studies.

Fourteen trials of predictive models for suspected CAD did not fulfill our inclusion criteria and were excluded because they consisted of unpublished abstracts (n=6), the populations did not meet our criteria (n=2), the model was not multivariate (n=1), or the risk scores were not validated (n=5) (Figure 1).

### Study characteristics and pertinent models

Ten studies 13-22 reporting the development of 12 models to predict CAD and two studies 4,23 focusing on external validation of models were included in the systematic review. A total of 53,108 patients (range 186 to 24,251 patients) were involved (not including Diamond 1979, which described an analysis using Bayes Theorem and did not specify the number of patients) (Table 1). The data sources for five of the models were multicenter trials. Only two of the models were developed using Asian populations, whereas the remainder were developed using Western populations. The outcomes of interest differed between models and represented substantial heterogeneity. These outcomes of interest included severe CAD in two models, functionally significant CAD in another model, and obstructive CAD in the remaining models. In terms of defining obstructive CAD, five models defined it as having at least one vessel with at least 50% diameter stenosis, while the remaining model defined it as having at least one vessel with ≥75% stenosis. The reference standards for diagnosing CAD included two procedures, ICA and coronary computed tomography angiography (CTA). Regarding model development methods, only one study used Bayesian-based algorithms, while the others used multivariable logistic regression analyses.

The number of predictors in each model ranged from three to 10 (Table 2). Nearly all of the diagnostic models included age, sex, and chest pain symptom as variables. Other common measures included in the majority of the risk models were diabetes mellitus, hypertension, smoking, and hyperlipidemia. Very few of the models included the hospital setting as a variable. Only one model included estrogen status or genetic profile as a risk factor.

### Model performance and validation

Seven models were validated internally, one with the bootstrap method, two with split samples 14,19, and four by cross-validation using the same population 18,20. Nine models reported the C statistic for the validation cohorts, which ranged from 0.49 to 0.88, indicating a degree of discriminative performance that varied from poor to excellent. Seven models were validated externally. Among those, four models 13-15,17 were reported in more than one external validation study, and their discriminating power showed a weakening trend in recent years (from 0.87 to 0.63). Only two studies reported calibration using the Hosmer-Lemeshow test for the derivation cohort, and both were adequate 18,21.The NRI was reported in one study 20 (Table 3).

Of the 12 models, only two had good discriminating power upon validation (C statistic >0.80). Eight and nine predictors were present in these two models. These two higher-performing models included the following risk factors: sex, age, symptoms (chest pain), diabetes status, smoking status, and hyperlipidemia status. In addition to the previously listed common risk factors, one model also included the coronary calcium score, hypertension status, and hospital setting, whereas the other included the ECG results and history of previous myocardial infarctions. Six models had moderate discriminative ability (C statistics of 0.70-0.80). These models included the following risk factors: chest pain symptoms (five models), diabetes or hyperlipidemia status (four models), hypertension or smoking status (three models), and hospital setting (two models). Other risk factors, such as history of cerebral infarction or peripheral vascular disease, were included only in one model. All models included sex and age as risk factors.

### Quality assessment and clinical usefulness

Important differences were present in the bias risk among the studies, with no single study satisfying all seven variables (Table 4). Although all studies adequately described subject selection and the certainty of the study outcomes, study attrition was described only in one of the studies. In addition, most of the studies on the 12 models provided little information on the selection (nine models) and measurement (seven models) of the prognostic factors.

As described above, information on model calibration and reclassification was absent from most studies. In addition, only two studies excluded patients with missing data, and the remaining studies did not report the amount of missing data. None of the studies reported the use of imputation techniques.

Half of the models (six of 12) stratified their cohorts into risk categories (low, moderate, and high risk). However, only one model explained how risk category assignment would affect diagnostic or therapeutic decisions. Simple risk calculators or web-based calculators were provided in three of the studies. One study evaluated the implications of the diagnostic model on clinical decision making and patient outcomes.

## DISCUSSION

In our systematic review, we identified 10 studies that described 12 diagnostic models for estimating the probability of CAD, and we found that these models yielded similar discriminative abilities. Only two of the models were assessed in clinical practice. Our study is the first to synthesize the available literature on predictive models of the PTP calculation for CAD, and it highlights the need for further development and refinement of these models.

### Model performance and validation

Only two of the models exhibited good discrimination based on the C statistic. However, one model included the coronary calcium score, which was assessed by CTA, as a risk factor. Because decisions on diagnostic testing are usually made before CTA is performed, inclusion of the coronary calcium score as a variable in the model limits its usefulness. The C statistic from the external validation of one of the other models showed a weakening trend (0.87 to 0.63) in recent years. An important cause of this trend is that the guidelines recommend using predictive models for PTP prior to non-invasive testing; therefore, this study chose CTA as the reference standard, whereas previous studies often regarded ICA as the reference standard. The model was developed using a population with a high prevalence of CAD who underwent ICA but was validated in a population with a low prevalence of CAD who underwent CTA. This caused the model to show poor discriminative ability. Indeed, an analysis restricted to patients who underwent ICA could be affected by verification bias. Other reasons for this phenomenon included that the entire population used to develop this model lived in the USA, and the model was developed 30 years ago. The differences in prevalence of risk factors and the homogeneity in some of the study populations limit the generalizability of these models. Therefore, a PTP assessment model should be carefully chosen based on the clinical characteristics of the population of interest.

Almost all of the models were developed using multivariable logistic regression analysis, but the performance of these models was unsatisfactory. Several studies have demonstrated that data mining is a novel and promising approach for enhancing the performance ability of a model. Green et al. 24 compared the abilities of artificial neural networks (ANN) and multiple logistic regression models to predict acute coronary syndrome in the emergency room. The results of this comparison showed that the C statistics of the best ANN ensemble and the best logistic regression model were 0.81 and 0.76, respectively (*p=0.03) 24. Similarly, Alizadehsani et al. 25 used data mining for the diagnosis of CAD and showed that characteristic chest pain, region RWMA2, and age were the most effective features, in addition to the features created using Information Gain. In addition, using this method and the feature creation algorithm, 94.08% accuracy was achieved, which is higher than current approaches in the literature 25. Future studies could use data mining to develop PTP calculation models for CAD.

Chest pain characteristics were incorporated into nearly all of the models as a risk factor. However, some studies show that chest pain categorization has a limited ability to predict substantial CAD. When patient symptoms are used to predict substantial CAD, dissociation frequently occurs between coronary atherosclerosis and ischemic heart disease. Angina can occur in the absence of obstructive lesions and in two of three patients with stable angina 26. Furthermore, women who undergo ICA due to chest pain are more likely to present with less extensive CAD than men. Women are also more likely to show evidence of myocardial ischemia and non-obstructive CAD 27. Therefore, using only symptoms as the main variables in the model may overestimate the probability of CAD, especially in women. Some variables specific to women should be considered during model development. Previous studies have demonstrated that adding gestational diabetes mellitus(GDM) and estrogen status to the Updated Diamond-Forrest model (UDFM) can result in a significant NRI (*p=0.04) 28. Our review included a model that incorporated estrogen status as a risk factor. Unfortunately, we found that this model performed poorly in the Middle Eastern cohort but was still better than the DFM, especially in symptomatic women 4,29.

### Use of diagnostic models in clinical practice

Although predictive models for the PTP of CAD have been a focus of research for approximately 10 years, acceptance by clinicians has been low. However, the ability to exclude clinically significant CAD using the PTP in even a small percentage of patients with normal coronary arteries could be very beneficial. According to a recent study, the rigid application of the NICE chest pain guidance, which is recommended for use in the DFM and DCS models for the PTP calculation, may result in up to two of three patients being excluded from further cardiac examinations 30. Several important factors may limit the application of these models in clinical practice. First, the risk factors included in the models are not readily available to physicians. For example, the DCS relies on the interpretation of an ECG and blood tests, whereas the DFM is more straightforward because it includes only information on age, sex, and symptoms. Therefore, compared to the DFM, it can be argued that the added variables in the DCS are not worth while because a more parsimonious risk assessment tool is most likely to be used in mainstream clinical practice 31. Second, unlike the Framingham risk score, almost all of the models in this review do not have an online calculator, which would make it difficult for doctors to calculate the PTP because they would need to use a complex formula. To enhance clinical utilization, the models should be based on demographic information and past histories that are typically accessible in real-world clinical practice. Equally important is the availability of electronic or web-based calculators to facilitate use at the bedside.

In addition, limited data are available regarding the use of these models in clinical practice and their effect on clinical decision making and cost-effectiveness. Only the DFM and DCS models were studied in a letter to the Editor. The report on these models showed that by following the standard diagnostic pathway, the total cost of examining all patients was £102,731, compared to £198,495 for the diagnostic pathway proposed by the NICE chest pain guidance 32.

### Clinical Implications

In general, our review could help clinicians better understand the development of diagnostic models for CAD and thus better determine the appropriate model to apply to their specific patient population. Clinicians should consider using one of the higher-performing models that include readily available risk factors. Accurate, simple diagnostic models are more easily accepted in clinical decision making processes and patient counseling. Additionally, this review could provide a reference for future modeling efforts. The differing definitions of CAD may affect the generalizability and calibration of the models, and thus further model development and validation studies with large cohorts and a wide variety of populations are needed. We should acknowledge that the present review has several limitations. First, the degree of stenosis in some of the studies was determined by quantitative coronary angiography, whereas other studies used visual assessment. Second, the studies included here involved heterogeneous populations and had differing definitions for CAD, which could have led to differential risks for CAD.

The available data indicate that current models for the PTP calculation of CAD are still not fully refined. Although we identified some higher-performing models, it is difficult to generalize these to populations beyond those included in the studies. Further research should focus on developing models for predicting CAD that are better suited to broader populations. Adequate clinical trials need to be conducted to assess the benefits of using these models in clinical practice for cases where CAD is not suspected.

## AUTHOR CONTRIBUTIONS

Ting H and Xing L are the main authors. Hong Y is the scientific adviser. Nana X, Ying L, Qiaoyu W and Meilin L provided assistance with the writing of the manuscript.

## Figures and Tables

**Table 1:** Characteristics of the predictive models for coronary heart disease.

Study	Country	Population and setting	Definition of CAD	Reference standard	Patients (No)		Predictions (No)	Events		Methods
					Derivation	Validation		Derivation	Validation	
Yang et al. (22)	An International Multicenter	Patients referred to CTA for suspected CAD	High-risk CAD was defined as left main diameter stenosis>50%, 3-vessel disease with diameter stenosis>70%, or 2-vessel disease involving the proximal left anterior descending artery	CTA	24,251	7,333	9 variables: age, sex, diabetes, hypertension, current smoking, hyperlipidemia, family history of CAD, history of peripheral vascular disease, and chest pain symptoms	877(3.6%)	349(4.8%)	multivariate logistic regression analysis
Fujimoto et al. (21)	Japanese population	Patients referred to CTA for suspected CAD	Obstructive CAD was defined as lesions with stenosis of 75% or more	CTA	4,137	319	8 variables: age, gender, hypertension, diabetes mellitus, dyslipidemia, smoking, history of cerebral infarction, and chest symptoms.	764(18.5%).	123(34.1%)	multivariate logistic regression analysis
Caselli et al. (20)	14 European centers	Patients with stable chest pain or equivalent symptoms and an intermediate probability of CAD	Functionally significant CAD was defined as an ICA causing myocardial ischemia at stress imaging or associated with reduced FFR<0.8, or both	CTA+ at least 1 coronary artery functional imaging test	527	186	6 variables:AST, hs-CRP levels, HDL cholesterol, symptom characteristics, age, sex	80(15.2%)	75(40%)	logistic regression analysis
Chen et al. (19)	China	Patients referred to ICA for suspected CAD	Severe CAD was defined as Gensini scores ≥20, which was approximately equal to onestenosed lesion ≥70% in the proximal left anterior descending artery	ICA	347	204	8 variables: age, sex, AVC, abnormal ECG, diabetes, hyperlipidemia, HDL, LDL	202(58.2%)	NR	logistic regression analysis
Genders et al. (18)	18 hospitals from Europe and the US	Patients were referred for catheter-based or CT-based coronary angiography	Obstructive CAD was defined as at least one vessel with at least 50% diameter stenosis on ICA	CTA+ICA	5,677	-	The basic model included 4 variables: age, sex, symptoms, and setting. Theclinical model included 8 variables: age, sex, symptoms, setting, diabetes, hypertension, dyslipidemia, and smoking, The extended model included 9 variables: age, sex, symptoms, setting, diabetes, hypertension, dyslipidemia, smoking, and coronary calcium score.	-	-	logistic regression analysis
Genders et al. (17)	14 hospitals from Europe and US	Patients presented with stable chest pain and an ICA was performed	Obstructive CAD was defined as ≥50% stenosis in one or more vessels on ICA	ICA	2,260	-	3 risk factors: sex, age, and symptoms	1,319(58.4%)	-	logistic regression analysis
Rosenberg et al. (16)	39 center prospective study	Non-diabetic patients who were referred for diagnostic ICA or had a high risk of CAD	Obstructive CAD was defined as ≥50% stenosis in ≥1 major coronary artery by quantitative coronary angiography	ICA	640	526	23 genes grouped in the 6 terms, 4 sex-independent and 2sex-specific	230(35.9%)	192(36.5%)	logistic regression analysis
Morise et al. (15)	US	Inpatients or outpatients were referred for exercise testing because of symptoms that raised the suspicion of coronary disease	Significant coronary artery disease was defined as the presence of ≥1 vessel with ≥50% luminal diameter narrowing	ICA	915	348	10 risk factors: sex, age, symptoms (chest pain), estrogen status, diabetes, hypertension, smoking, hyperlipidemia, family history, and obesity.	373 (41%)	157 (45%)	multivariate logistic regression analysis
Pryor et al. (14)	US	Patients with chest pain were referred for ICA	Significant coronary artery disease was defined as >75% luminal diameter narrowing of at least one major coronary artery	ICA	3,627	1,811	8 risk factors: sex, age, symptoms(chest pain), diabetes, smoking, hyperlipidemia, history of MI, and electrocardiogram (Q waves, ST-T wave changes)	2,379(65.6%)	1,266(69.9%)	multivariate logistic regression analysis
Diamond and Forrester(13)	US	Pooled analysis from 18 other studies	Obstructive CAD was defined as ≥50% stenosis in one or more vessels on CCA	coronary angiography	-	-	3 risk factors: sex, age, and symptoms	-	-	Bayesian-based algorithms

**Abbreviations:** CAD, coronary artery disease; CTA, coronary computed tomography angiography; ICA, invasive coronary arteriography; AST, aspartate aminotransferase; hs-CRP, high - sensitivity C-reactive protein; HDL, high-density lipoprotein; FFR, fractional flow reserve; AVC, aortic valve calcification; LDL, low-density lipoprotein; MI, myocardial infarction

**Table 2:** Variables included in the predictive models of pre-test probability of coronary heart disease.

Variable	Study (sample size)											
	Yang et al. (22)	Fujimoto et al. (21)	Caselli et al. (20)	Chen et al. (19)	Genders et al. (1) (18)	Genders et al. (2) (18)	Genders et al. (3) (18)	Genders et al. (17)	Rosenberg et al. (16)	Morise et al. (15)	Pryor et al. (14)	Diamond and Forrester (13)
**Demographic**												
Age	√	√	√	√	√	√	√	√		√	√	√
Sex	√	√	√	√	√	√	√	√		√	√	√
**Medical history**												
Diabetes mellitus	√	√		√		√	√			√	√	
Hypertension	√	√				√	√			√		
Previous MI											√	
Hyperlipidemia	√	√		√		√	√			√	√	
Cerebral infarction		√										
Peripheral vascular disease	√											
**Clinical presentation/physical examination**												
Chest pain	√	√	√		√	√	√	√		√	√	√
Coronary calcium score							√					
AVC				√								
Abnormal ECG				√							√	
**Personal history**												
Obesity										√		
Smoking	√	√				√	√			√	√	
Family history of CAD	√									√		
**Laboratory values**												
AST			√									
hs-CRP			√									
LDL				√								
HDL			√	√								
Estrogen status										√		
**Others**												
Hospital setting					√	√	√					
Genes									√			

**Abbreviations:** MI, myocardial infarction; AVC, aortic valve calcification; AST, aspartate aminotransferase; hs-CRP, high-sensitivity C-reactive protein; LDL, low-density lipoprotein; HDL, high-density lipoprotein

**Table 3:** Metrics of the performances of the models in predicting coronary heart disease.

Derivation cohort			Validation cohort(internal)			Validation cohort(external)			NRI value
Study	Population	Sample size, discrimination, calibration	Study	Type of internal validation	Sample size, discrimination, calibration	Study	Population	Sample size, discrimination, calibration	
Yang et al. (22)	Patients referred to coronary CTA for suspected CAD	N=24,251;0.76;NR	-	-	-	same	An additional non overlapping cohort of 7,333 patients	N=7,333;0.71;NR	
Fujimoto et al. (21)	Patients referred to coronary CTA for suspected CAD	N=4,137;0.74;0.74	-	-	-	same	Independent sample	N=319;0.71;NR	
Caselli et al. (20)	Patients with stable chest pain or equivalent symptoms and an intermediate probability of CAD	N=527;0.70;NR	same	cross-validation	N=527;0.66;0.80	same	An independent population of patients with suspected CAD	N=186;0.72;NR	0.15 *p*=0.041
Chen et al. (19)	551 patients with stable angina who were admitted for coronary angiography	N=347;0.74;NR	same	split sample	N=204;0.71; 0.43	-	-	-	-
Genders et al. (18)	Patients presented with stable chest pain and were referred for catheter-based or CT-based coronary angiography.	N=5,677;0.77/0.79/0.88;NR/NR/NR	same/ same/ same	cross-validated	N=3,955;0.78-0.81;0/0.35/1.2	-	-	-	
Genders et al. (17)	Patients presented with stable chest pain, and CCA was performed.	N=2,260;0.82; <0.001(men),0.02(women)	-	-	-	same/Yang 2015/Caselli 2015	Independent data set consisting of outpatients who presented with stable chest pain	N=471;0.76;NR/ N=24,251;0.64;NR/ N=527;0.63;NR	
Rosenberg et al. (16)	Non-diabetic patients with a history of chest pain, suspected angina-equivalent symptomsor a high risk of CAD who were referred for diagnostic ICA	N=640;0.70;NR	same	bootstrap	N=526;NR;<0.001	-	-	-	-
Morise et al. (15)	Inpatients or outpatients were referred for exercise testing because of symptoms that raised a suspicion of coronary disease.	N=918;NR;NR	-	-	-	same/Isma'eel 2015	Patients were referred for exercise testing or coronary angiography because of suspected coronary disease/Middle Eastern patients with chest pain were referred for coronary angiography.	N=348;NR;NR/ N=378;0.61(men), 0.58(women);NR/	-
Pryor et al. (14)	5,438 patients with chest pain were referred for cardiac catheterization.	N=3,627;NR;NR	same	split sample	N=1,811; specificity 0.33; sensitivity 0.94;NR	same/ Pryor 1993/ Genders 2012/ Fujimoto 2015	CASS population and pooled estimates reported by Diamond 1979/Outpatients underwent catheterization/Patients presented with stable chest pain and were referred for catheter-based or CT-based coronary angiography.	NR;NR;NR\ N=168;0.87;NR\ N=4,426;0.78;0.001\ N=527;0.70;NR	
Diamond and Forrester (13)	Pooled analysis from 18 other studies	N=28,948;NR;NR	-	-	-	Rosenberg 2010/Genders 2011/ Chen 2014/ Ismaeel 2015	Non-diabetic patients from multiple centers/Patients presented with stable chest pain/ Chinese patients with angina/Middle Eastern patientswith chest pain	N=526;0.66;NR/ N=1,683;NR;NR/ N=377;0.73;NR/ N=378; 0.59(men), 0.49(women);NR	

**Abbreviation:** CAD, coronary artery disease; CTA, computed tomography coronary angiography; ICA, invasive coronary arteriography; NR: notreported

**Table 4:** Guidelines for assessing the quality of prognostic studies based on the framework of potential biases.

**Study**	Potential Bias							Clinical Usefulness	
	Study participation	Study attrition	Prognostic factor selection	Prognostic factor measurement	Outcome measurement	Statistical analysis	Reporting of model performance	Utility	Usability
Yang et al. (22)	Low	?	Low	High	Low	Low	Low	Yes	No
Fujimoto et al. (21)	Low	?	Low	?	Low	Low	Low	No	No
Caselli et al. (20)	Low	?	Low	Low	Low	Low	Low	No	No
Chen et al. (19)	Low	?	Low	?	Low	Low	Low	Yes	No
Genderset al. (18)	Low	?	Low	Low	Low	Low	Low	Yes	Yes
Genders et al. (17)	Low	?	Low	High	Low	Low	Low	Yes	Yes
Rosenberg et al. (16)	Low	?	?	Low	Low	Low	Low	No	No
Moriseet al. (15)	Low	?	Low	?	Low	Low	High	Yes	No
Pryor et al. (14)	Low	Low	Low	?	Low	Low	High	Yes	No
Diamond and Forrester (13)	High	?	?	?	Low	Low	High	No	Yes
